# RETRACTION: Effect of Transverse Colostomy Versus Ileostomy in Colorectal Anastomosis on Post‐Operative Wound Complications: A Meta‐Analysis

**DOI:** 10.1111/iwj.70392

**Published:** 2025-03-21

## Abstract

**RETRACTION:** ZhangQ. , LiuF. , LiY. , JiL. , YuY. , and YangX. , “Effect of Transverse Colostomy Versus Ileostomy in Colorectal Anastomosis on Post‐Operative Wound Complications: A Meta‐Analysis,” International Wound Journal 21, no. 3 (2024): e14428, 10.1111/iwj.14428.37938886 PMC10895195

The above article, published online on 08 November 2023, in Wiley Online Library (http://onlinelibrary.wiley.com/), has been retracted by agreement between the journal Editor in Chief, Professor Keith Harding; and John Wiley & Sons Ltd. Following an investigation by the publisher, all parties have concluded that this article was accepted solely on the basis of a compromised peer review process. The editors have therefore decided to retract the article. The authors did not respond to our notice regarding the retraction.

**RETRACTION:** Q. Zhang , F. Liu , Y. Li , L. Ji , Y. Yu , and X. Yang , “Effect of Transverse Colostomy Versus Ileostomy in Colorectal Anastomosis on Post‐Operative Wound Complications: A Meta‐Analysis,” International Wound Journal 21, no. 3 (2024): e14428, 10.1111/iwj.14428.37938886 PMC10895195

The above article, published online on 08 November 2023, in Wiley Online Library (http://onlinelibrary.wiley.com/), has been retracted by agreement between the journal Editor in Chief, Professor Keith Harding; and John Wiley & Sons Ltd. Following an investigation by the publisher, all parties have concluded that this article was accepted solely on the basis of a compromised peer review process. The editors have therefore decided to retract the article. The authors did not respond to our notice regarding the retraction.

